# Hypothetical Atopic Dermatitis-Myeloproliferative Neoplasm Syndrome

**DOI:** 10.3389/fimmu.2015.00434

**Published:** 2015-08-24

**Authors:** Toshiaki Kawakami, Tomoaki Ando, Yuko Kawakami

**Affiliations:** ^1^Division of Cell Biology, La Jolla Institute for Allergy and Immunology, La Jolla, CA, USA; ^2^Laboratory for Allergic Disease, RIKEN Center for Integrative Medical Sciences (IMS-RCAI), Yokohama, Japan

**Keywords:** atopic dermatitis, myeloproliferative neoplasm, hematopoietic stem cell, mast cell, mouse models

## Abstract

Atopic dermatitis (AD) is a chronic inflammatory skin disease. Myeloproliferative neoplasms (MPNs) are hematopoietic malignancies caused by uncontrolled proliferation of hematopoietic stem/progenitor cells. Recent studies have described several mutant mice exhibiting both AD-like skin inflammation and MPN. Common pathways for skin inflammation encompass overexpression of thymic stromal lymphopoietin and reduced signaling of epidermal growth factor receptor in the epidermis, while overproduction of granulocyte-colony-stimulating factor by keratinocytes and constitutive activation of Stat5 in hematopoietic stem cells are important for the development of MPN. The murine studies suggest the existence of a similar human disease tentatively termed as the atopic dermatitis-myeloproliferative neoplasm syndrome.

Atopic dermatitis (AD) is a chronic pruritic inflammatory skin disease. Although the etiology of AD is not completely understood, clinical and basic studies suggest that impaired skin barrier function and immune dysregulation underlie the disease (1). For example, loss-of-function mutations in the filaggrin gene (*FLG*) are the most well-characterized risk factor for the development of AD (2, 3). Filaggrin plays a critical role in skin barrier function (4). Tight junctions below the stratum corneum also contribute to skin barrier. The expression of the tight junction proteins, claudin-1 and claudin-23, is reduced in patients with AD (5). In immunological terms, AD has been characterized by excessive Th2 cell and eosinophilic infiltration in acute lesions and a mixed Th1 and Th2 pattern in chronic lesions (6). Recent studies also suggest the involvement of Th17 cells in the acute but not the chronic phase (7, 8). Additionally, the expression of genes encoding neutrophil chemoattractants was revealed in AD lesions, consistent with increased neutrophil infiltration in the lesions (9).

Mechanistic studies of AD have been mostly conducted by mouse experiments (10, 11) despite perceived differences in human and mouse biology (12–14). Importantly, several mutant mice develop both AD-like skin inflammation and myeloproliferative neoplasm (MPN), suggesting the existence of a disease tentatively termed as the atopic dermatitis-myeloproliferative neoplasm (AD-MPN) syndrome. A majority of AD models do not develop MPN, as AD is a common disease but MPN is a rare disease. MPNs are hematopoietic malignancies characterized by an overproduction of any combination of white cells, red cells, and platelets. Human MPNs include polycythemia vera, essential thrombocythemia, primary myelofibrosis, and chronic myelogenous leukemia (15). Interestingly, pruritus is a common symptom in Philadelphia chromosome-negative MPNs. Suggestive of the role of mast cells in both disease phenotypes, mast cells from MPNs release greater amounts of pruritogenic factors, such as histamine, leukotrienes, and IL-31 (16). The mutant mice with epidermal deletion [by K5 (or K14)-Cre-mediated deletion of a floxed gene] of JunB (transcription factor) (17), a disintegrin and metalloproteinase 17 (ADAM17) (18, 19) or Notch receptors/their signal transducers (20) exhibited both AD-like skin inflammation and MPN. We recently described AD-like dermatitis and MPN in phospholipase C (PLC)-β3-deficient mice (21, 22). Similar skin inflammation accompanied by increased serum IgE and IgG1 was also described with mice deficient in *SOCS7*, a negative regulator of cytokine action by inhibiting transcription factors Stat5 and Stat3 (23). Therefore, in these mutant mice, AD and MPN pathologies likely develop by the shared or overlapping molecular and cellular mechanisms.

Myeloproliferative neoplasms are caused by uncontrolled proliferation and/or survival of hematopoietic stem/progenitor cells (24). Mechanistically, myeloproliferation could be caused by granulocyte-colony-stimulating factor (G-CSF) produced by JunB-deficient keratinocytes in *JunB^fl/fl^;K5-Cre* mice (17) and ADAM17-deficient keratinocytes in *Adam17^fl/fl^;K14-Cre* mice (Figure 1) (18). Consistent with the crucial role for keratinocyte-derived G-CSF, the loss of G-CSF *in vivo* prevented MPN and reduced skin inflammation in *JunB^fl/fl^;K5-Cre* mice. Transplantation of bone marrow cells derived from *JunB^fl/fl^;K5-Cre* mice failed to transfer of the MPN phenotype to WT mice. JunB is a direct repressor of *Csf3* (gene encoding G-CSF) transcription (17). ADAM17 cleaves membrane proteins, working as the principal sheddase for tumor necrosis factor and several ligands of the epidermal growth factor receptor (EGFR). ADAM17 also activates Notch in adult epidermis. In *Adam17*^−^*^/^*^−^ mice, impaired Notch signaling (i.e., decreased Notch intracellular domain) resulted in increased c-Fos recruitment to the *Csf3* promoter leading to increased *Csf3* transcription, as Notch signaling antagonized c-Fos recruitment to the *Csf3* promoter (18). G-CSF might also be involved in skin inflammation by promoting keratinocyte proliferation (25). Consistent with the role of reduced EGFR signaling in AD-like skin inflammation in *Adam17^fl/fl^*;*K14-Cre* mice, an ADAM17-targeted EGFR ligand transforming growth factor-α, whose expression was strongly reduced in *Adam17*^−^*^/^*^−^keratinocytes, could reconstitute the skin barrier function in *Adam17*^−^*^/^*^−^ mice, and mice with epidermal deletion of EGFR exhibited AD-like dermatitis just like *Adam17^fl/fl^*;*K14-Cre* mice (19). Another common pathway for skin inflammation might be overexpression of thymic stromal lymphopoietin (TSLP) (Figure 1), an IL-7-like cytokine produced by keratinocytes (26, 27), in lesional epidermis (18, 22, 28). Notch signaling also antagonized c-Fos recruitment to the *Tslp* promoter (18). Skin-specific overexpression of TSLP in transgenic mice resulted in the development of eczematous lesions and elevated serum levels of IgE (29, 30). Both spontaneous and allergen-induced dermatitis in *Plcb3*^−^*^/^*^−^mice required the receptor for TSLP (31). Expression of TSLP was extremely high in lesional epidermis of *Plcb3*^−^*^/^*^−^ mice. TSLP could directly activate cutaneous sensory neurons, triggering itch (32), a major symptom that impairs the quality of life in AD patients. Mast cells, which were required for spontaneous and allergen-induced AD models (22, 31), were increased in lesional skin of *Plcb3*^−^*^/^*^−^mice due to the increased Stat5 and reduced SHP-1 (protein tyrosine phosphatase) activities (Figure 1) (22). PLC-β3 functions as an adaptor by directly binding to SHP-1 and Stat5, and Stat5 activity is inactivated by dephosphorylation of Stat5–Tyr694 by SHP-1 (21, 33). Consistent with the role of Stat5 in AD and MPN, itchy dermatitis with increased mast cells developed in transgenic mice expressing Jak2 V617F, an activating mutation found in many human MPN patients that also caused a polycythemia vera-like disease in the transgenic mice (34). The STAT5-regulatory mechanism for increased mast cells seemed important for AD pathogenesis in human patients as well (22). Breakdown of the same Stat5-regulatory mechanism was also responsible for the uncontrolled growth of hematopoietic stem cells (HSCs) in MPN in *Plcb3*^−^*^/^*^−^mice (21).

**Figure 1:**
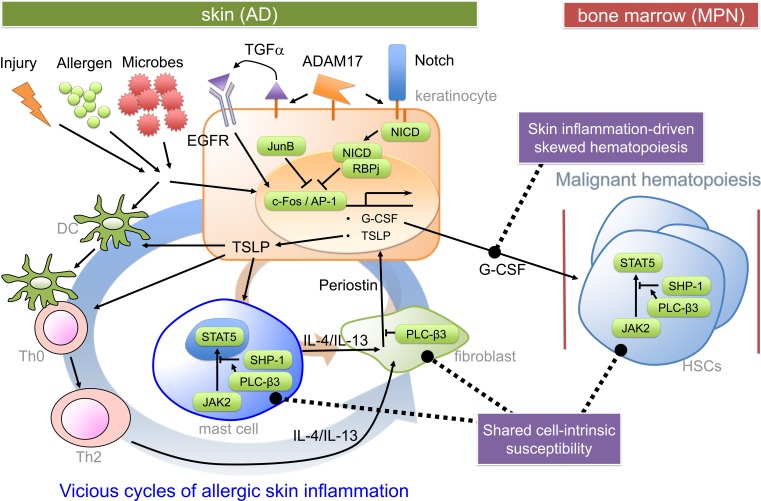
**Pathogenic mechanisms for the hypothetical AD-MPN syndrome**. Keratinocyte-derived TSLP (and impaired skin barrier) seems to play a critical role in dendritic cell-dependent Th2 responses to offending allergens or injury. Th2 cytokines predominantly cause an acute inflammation and activation of several types of cells, including dermal fibroblasts. Th2 cytokine-stimulated fibroblasts secrete periostin, an α_v_ integrin-interacting matricellular protein, which in turn stimulates keratinocytes to secrete TSLP (35). TSLP also stimulates mast cells to secrete Th2 and other cytokines. Periostin secretion from dermal fibroblasts depends on Th2 cytokines and mast cells, forming at least two positive feedback loops (indicated thick and thin circular loops). Keratinocyte-derived G-CSF is important for MPN development in certain mutant mice. Expression of genes coding for G-CSF and TSLP is under control of c-Fos/AP-1, which is positively regulated by EGFR and negatively regulated by Notch signaling and JunB. The proliferative and survival properties of hematopoietic stem cells (HSCs) are positively regulated by Stat5, whose constitutive activation can lead to MPN. PLC-β3 negatively regulates TSLP production in keratinocytes, periostin production in fibroblasts, and Stat5 activity in HSCs, mast cells, and Th2 cells. DC, dendritic cell; NICD, Notch intracellular domain; RBPj, recombination signal binding protein for immunoglobulin kappa region.

Are allergic diseases such as AD protective or causative for the hematopoietic malignancies? Behind this question, there are two hypotheses to explain positive versus negative associations of the atopic diseases to malignancy: the “immunosurveillance” theory proposes that the atopic inflammatory state stimulates the immune system to eliminate the transformed cells. On the contrary, in the “antigenic stimulation” theory, the atopic or immune-stimulating condition leads to randomly occurring pro-oncogenic mutations in actively proliferating cells (36). Some prospective cohort studies support the hypothesis that the allergic diseases could be the risk of hematopoietic malignancies (leukemia, lymphoma, and myeloma), prostate cancer, and breast cancer (36–43), suggesting that specific types of allergies might increase risks for certain malignancies. But ovarian cancer risk was decreased in allergic patients (38). However, a meta-analysis of several case–control studies reported no increased risk of leukemia (44).

The hypothetical AD-MPN syndrome might have similarities with malignancy-associated Sweet’s syndrome or acute febrile neutrophilic dermatosis (45). Sweet’s syndrome occurs most often associated with acute myelogenous leukemia and is characterized by fever, neutrophilia, erythematous skin lesions, and a diffuse infiltrate consisting predominantly of mature neutrophils (i.e., these skin lesions are different from typical AD lesions). Thus, acute myelogenous leukemia-associated Sweet’s syndrome might be an acute evolution of an insidious MPN. Moreover, some Sweet’s syndrome patients have mutations in the *PTPN6* gene encoding SHP-1 (46) (Figure 1). Interestingly, TSLP receptor (also known as CRLF2) overexpression has been associated with certain subtypes of pediatric leukemia (47, 48). Therefore, it will be interesting to investigate whether humans have an AD-MPN syndrome.

## Author Contributions

TK, TA, and YK all contributed to write the manuscript. TA prepared the figure.

## Conflict of Interest Statement

The authors declare that the research was conducted in the absence of any commercial or financial relationships that could be construed as a potential conflict of interest.

## Abbreviations

AD, atopic dermatitis; EGFR, epidermal growth factor receptor; G-CSF, granulocyte-colony-stimulating factor; MPN, myeloproliferative neoplasm; PLC, phospholipase C; TSLP, thymic stromal lymphopoietin.
